# Early and late CTCAE and patient-reported outcomes following breast radiotherapy in the REQUITE cohort

**DOI:** 10.1016/j.ctro.2026.101307

**Published:** 2026-10-07

**Authors:** Tina Bockstiegel, Adam Webb, Miguel E. Aguado-Barrera, Ester Aguado-Flor, Manuel Altabas, Marianne Aznar, David Azria, Erik Briers, Renée Bultijnck, Jenny Chang-Claude, Alessandro Cicchetti, Maria Carmen De Santis, Alison Dunning, Laura Fachal, Olivia Fuentes-Rios, Alexandra Giraldo, Antonio Gómez-Caamaño, Sara Gutiérrez-Enríquez, Carsten Herskind, Philipp Heumann, Kerstie Johnson, Rudolf Kaaks, Sarah L. Kerns, Maarten Lambrecht, Eliana La Rocca, Meritxell Mollà, Tiziana Rancati, Victoria M. Reyes, Juan Camilo Rosas Romero, Barry S. Rosenstein, Dirk De Ruysscher, Petra Seibold, Rebecca M. Shearer, Hilary Stobart, R. Paul Symonds, Begoña Taboada-Valladares, Christopher J. Talbot, Ana Vega, Liv Veldeman, Marlon R. Veldwijk, Tim Ward, Catharine M.L. West, REQUITE/REQUITEplus/RADprecise consortia, Tim Rattay, Elena Sperk

**Affiliations:** a Mannheim Comprehensive Cancer Center, Universitätsmedizin Mannheim, Medical Faculty Mannheim, Heidelberg University, Mannheim, Germany; b Department of Radiation Oncology, Universitätsmedizin Mannheim, Medical Faculty Mannheim, Heidelberg University, Mannheim, Germany; c University of Leicester, United Kingdom; d Grupo Genética en Cáncer y Enfermedades Raras, Instituto de Investigación Sanitaria de Santiago de Compostela, Santiago de Compostela, Spain; e Fundación Pública Galega de Medicina Xenómica, Santiago de Compostela, Spain; f Hereditary Cancer Genetics Group, Vall d'Hebron Institute of Oncology (VHIO), Vall d'Hebron Barcelona Hospital Campus, Barcelona, Spain; g Programa de Doctorat en Biomedicina, Universitat de Barcelona (UB), Barcelona, Spain; h Department of Radiation Oncology, Vall d'Hebron Hospital Universitari, Vall d'Hebron Barcelona Hospital Campus, Barcelona, Spain; i University of Manchester, Christie Hospital NHS Foundation Trust, United Kingdom; j Fédération Universitaire d'Oncologie Radiothérapie d'Occitanie Méditerranée, ICM, Univ Montpellier INSERM U1194 IRCM, Montpellier, France; k Patient Advocate, United Kingdom; l Department of Human Structure and Repair, Ghent University, Ghent, Belgium; m Division of Cancer Epidemiology, German Cancer Research Center (DKFZ), Heidelberg, Germany; n Fondazione IRCCS Istituto Nazionale dei Tumori, Milan, Italy; o University of Cambridge, United Kingdom; p Wellcome Sanger Institute, United Kingdom; q Department of Radiation Oncology, Universitätsmedizin Mannheim, Medical Faculty Mannheim, Heidelberg University, Mannheim, Germany; r Medical Faculty Heidelberg, Heidelberg University, Germany; s Leicester Cancer Research Centre, University of Leicester, Leicester, UK; t Medical College of Wisconsin, USA; u UZ Leuven, Belgium; v Department of Radiation Oncology, Fondazione IRCCS Istituto Nazionale dei Tumori, Milan, Italy; w Radiation Oncology Department, Hospital Clinic of Barcelona, Spain; x Departments of Radiation Oncology & Genetics and Genomic Sciences, Icahn School of Medicine at Mount Sinai, New York, USA; y MAASTRO Clinic, Maastricht, Netherlands; z Division of Personalized Early Detection of Prostate Cancer, German Cancer Research Center (DKFZ), Heidelberg, Germany; aa The Christie NHS Foundation Trust, United Kingdom; ab Department of Radiation Oncology, Hospital Clínico Universitario de Santiago de Compostela, Servizo Galego de Saúde (SERGAS), Santiago de Compostela, Spain; ac Ghent University Hospital, Belgium

**Keywords:** Breast cancer, Radiotherapy, Adverse events, Prospective cohort study, Patient-reported outcomes, REQUITE, Fractionation

## Abstract

**Purpose:**

The international REQUITE study and its follow-up studies (RADprecise/REQUITEplus) prospectively collected longitudinal data from patients undergoing radiotherapy for breast, prostate, or lung cancer. We present data from 2057 women with breast cancer, describing patient-reported outcomes and acute and late adverse effects.

**Methods:**

Patients were recruited between April 2014 and October 2016. Eligible patients received external-beam photon radiotherapy after breast-conserving surgery, had no metastasis at inclusion, no other malignancy within the previous five years, and no prior breast irradiation. Blood samples were collected before radiotherapy for genotyping, molecular analysis, and biobanking. Standardised assessments included CTCAE v4, EORTC QLQ-C30 and BR23, MFI, GPAQ, and DICOM/DVH data. Follow-up continued annually for two years, extending to eight years at eligible sites.

**Results:**

Acute side effects peaked at end of radiotherapy, with erythema in 83–89% of patients. Grade ≥ 2 acute effects, including pain (11% versus 4.3%) and skin ulceration (4.8% versus 0.8%), were more frequent with normofractionation than hypofractionation. Grade ≥ 2 late effects affected between 0.6% and 16.8% of patients; skin induration was the most frequent late effect. Patient-reported outcome scores varied over time, with functional scale scores at two years similar to or higher than at baseline.

**Conclusion:**

REQUITE provides real-world evidence of acute and late outcomes following normo- and hypofractionation, broadly consistent with published randomised trials. The dataset, combining clinical adverse effects, quality of life, dosimetric, and genetic data, is a resource for personalised radiotherapy research (www.requite.eu).

## Highlights

• Multi-national cohort (*n* = 2057) of breast cancer patients with RT.
• Long-term patient-reported outcomes and adverse effects data up to 8 years.
• Acute adverse effects peaked at end of RT and declined thereafter.
• Descriptive findings on late adverse effects broadly consistent with randomised trials.
• Real-world evidence combining PROs, CTCAE, DICOM/DVH and blood samples across multiple centres.

## 1 Introduction

Breast cancer is the most common form of malignancy among women worldwide [1]. The optimal treatment approach is determined by multiple factors that reflect both the disease characteristics and patient-specific considerations. Key determinants include tumour stage, size, and the clinical presentation of the patients, along with the presence or absence of molecular markers such as HER2 status, hormone receptor status and other molecular markers. Treatment commonly involves surgery followed by radiotherapy but may be further complemented by endocrine therapy, systemic chemotherapy and/or other targeted therapies. Each therapeutic approach is carefully tailored to the individual patient to maximize efficacy and minimize adverse effects [2].

Personalised oncology is not limited to drug administration and surgical approaches but also applies to radiotherapy. In addition to personalised radiation plans, which have been individually calculated for decades, there are now various fractionation schedules and techniques for minimising the dose in organs-at-risk such as the heart and lungs, for example through deep-inspiration breath-hold or different positioning such as prone positioning. The target volume can also be changed, so that in special situations partial breast irradiation can be used instead of whole-breast irradiation to minimize adverse effects [3]. Reliable, longitudinal data on acute and late adverse effects and patient-reported outcomes, across both early and late follow-up time points and by fractionation schedule, are essential to inform these individualised treatment decisions.

The REQUITE study, along with the subsequent REQUITEplus and RADprecise projects, aimed to systematically investigate clinical and treatment data to better understand individual variability in radiotherapy outcomes. To enable biomarker analyses, including genetic profiling, blood samples were collected and stored in a central biobank, while comprehensive patient and treatment data were recorded through standardised case report forms.

Here we present data from the breast cancer patient cohort in the REQUITE study and its follow-up studies REQUITEplus/RADprecise, extending up to eight years after radiotherapy in some participants. Data is available on request (http://www.requite.eu; https://www.dkfz.de/en/cancer-epidemiology/radprecise) and can be used for different research projects, including genotyping, adverse effects, cosmetic outcome, patient reported outcomes, treatment data and radiotherapy planning data (DVHs, DICOM). Data on the prostate and lung cancer cohorts of the REQUITE study are published as part of an article series.

Data from the REQUITE breast cancer cohort have already been used in several studies, demonstrating the ongoing usefulness of this resource (https://pubmed.ncbi.nlm.nih.gov/?term=requite+breast). The aim of this paper is to provide a detailed, longitudinal description of CTCAE-graded acute and late adverse effects and patient-reported outcomes in this cohort, thereby complementing the existing resource and supporting future studies on personalised radiotherapy.

## 2 Methods

### 2.1 Study design

From April 2014 to October 2016, women aged 18 or over diagnosed with breast cancer were recruited into the REQUITE study (ISRCTN98496463) following breast-conserving surgery +/−chemotherapy prior to the start of adjuvant radiotherapy. Exclusion criteria were other malignancy within the previous five years, male sex, prior breast radiation, planned use of protons, concomitant chemo-radiation, mastectomy, bilateral breast cancer, pregnant patients, partial breast irradiation and patients with breast implants if not removed during surgery. Patients were recruited at 27 centres in Belgium, France, Germany, Italy, Spain, the United Kingdom (UK), and the United States (US) and received curative external-beam radiotherapy according to local tumour board recommendations and national/international guidelines. Prior to radiotherapy (and any chemotherapy if indicated), participants were invited to donate blood samples for DNA genotyping, RNA molecular marker assessment, and future storage. Detailed information regarding the study protocol and assessment of biomaterial has already been published [4]. All centres participated in the REQUITE study for a period of two years. Further follow-up depended on whether the centre participated in the REQUITEplus or RADprecise studies (DRKS00020290) which extended the follow-up of the patients participating in the REQUITE study up to eight years following radiotherapy. Owing to small patient numbers at individual later timepoints, follow-up beyond five years is reported as a combined 5–8-year category (Fig. 1). Detailed data for each timepoint are available for further research on request.

**Fig. 1:**
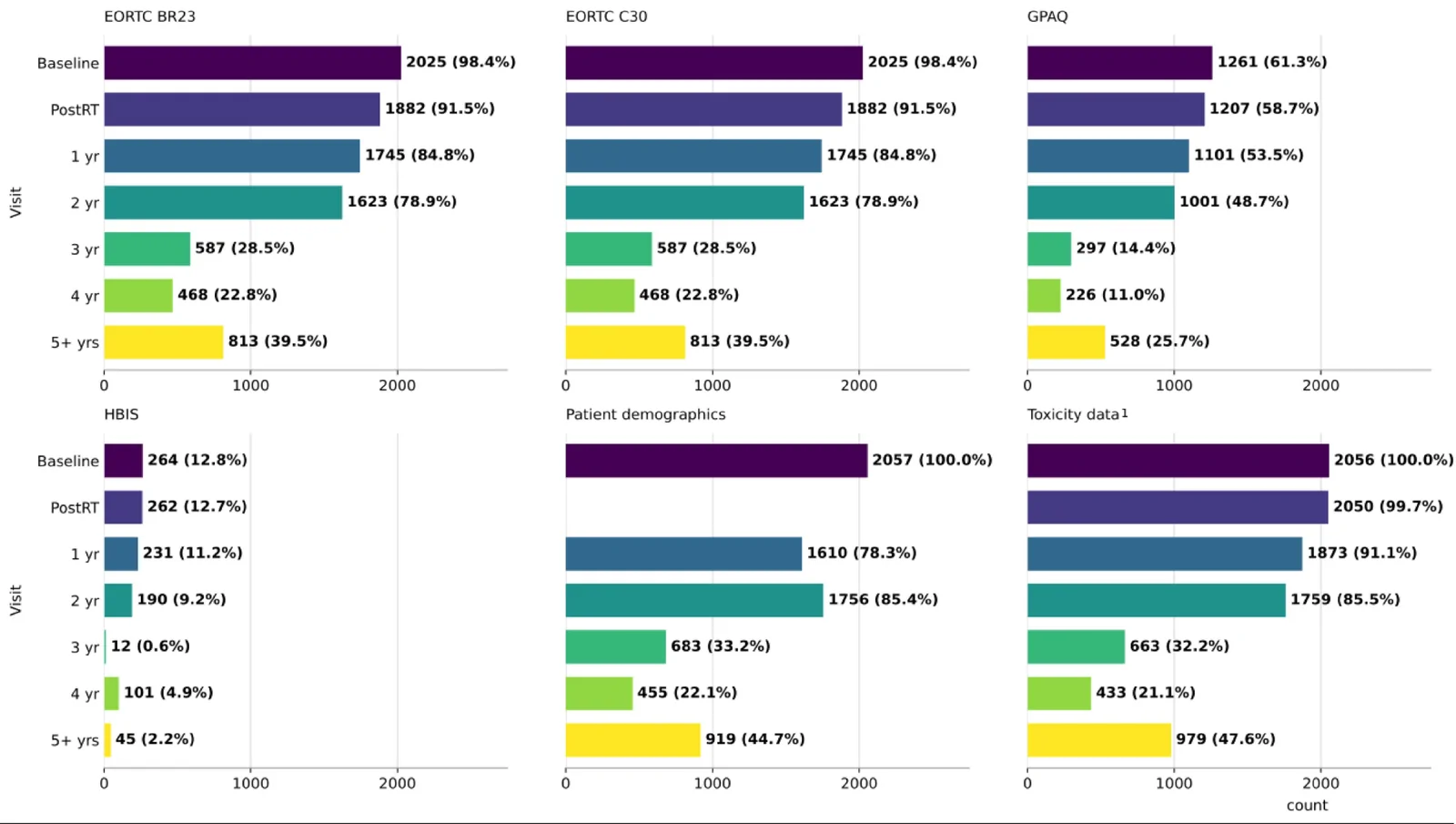
eCRFs and PROs collected by timepoint in absolute and relative frequencies. CTCAE dataPercentages are calculated relative to the total enrolled cohort (N=2,057); of note, [GPAQ/HBIS] was not administered at all participating sites, so completion rates among eligible patients are higher than the percentages shown.For the combined 5–8-year category, the maximum grade recorded for each patient within this period is shown.

These studies were approved by local ethical committees and all participants provided written informed consent for each study.

### 2.2 Data collection and radiotherapy information

For the standardised CRFs, patient factors baseline questionnaire was administered before radiotherapy initiation and at each follow-up, in which comorbidities, medication, lifestyle factors such as alcohol and nicotine consumption, and demographic patient data were recorded. Patient-reported outcomes (PROs) were assessed using standardised questionnaires administered at baseline before radiotherapy initiation but after surgery, at the end of radiotherapy (±5 days), and annually thereafter. The European Organization for Research and Treatment of Cancer (EORTC) QLQ-C30 questionnaire [5] was used to evaluate general symptoms, functioning, and quality of life, while the EORTC QLQ-BR23 [6] assessed breast cancer-specific symptoms including body image, sexual functioning, arm symptoms, breast symptoms, and systemic therapy side effects. Data on fatigue dimensions using the Multidimensional Fatigue Inventory (MFI) [7] questionnaire and general activity using the Global Physical Activity Questionnaire (GPAQ) [8] were collected in a sub-cohort (not collected in Belgium). Additionally, body image concerns were assessed using the Hopwood Body Image Scale (HBIS) [9], exclusively administered in the UK . Side-effects were assessed using CTCAE v4 [10]. Specific grading definitions are provided in Table 2. Cancer treatment data and radiotherapy physics data (e.g. DICOM-RT files) are available for almost all patients.

### 2.3 Statistical methods

All analyses were purely descriptive; no formal statistical hypothesis testing was performed. Cohort characteristics, including age, genetic predictors, and treatment, are presented in Supplementary Table 1 overall and in Table 1 stratified by dose per fraction.

**Table 1:** Characteristics of REQUITE and RADprecise breast cancer patients at baseline prior to radiotherapy stratified by dose per fraction.

Characteristic	Overall	≤ 2.2 Gy	> 2.2 Gy
	*N* = 2056^a^	*N* = 1062^a^	*N* = 994^a^
Country			
Belgium	550 (27%)	107 (10%)	443 (45%)
France	453 (22%)	415 (39%)	38 (3.8%)
Germany	212 (10%)	201 (19%)	11 (1.1%)
Italy	102 (5.0%)	82 (7.7%)	20 (2.0%)
Spain	315 (15%)	169 (16%)	146 (15%)
United Kingdom	348 (17%)	30 (2.8%)	318 (32%)
United States	76 (3.7%)	58 (5.5%)	18 (1.8%)
Age (years)	58 (50, 66)	55 (48, 64)	61 (53, 69)
Weight at cancer diagnosis (kg)	68 (60, 78)	66 (58, 76)	69 (61, 79)
Unknown	14	6	8
Height (cm)	163 (158, 168)	163 (159, 168)	163 (157, 168)
Unknown	10	3	7
Smoker			
Never	1155 (57%)	589 (56%)	566 (58%)
Ex before cancer diagnosis	514 (25%)	249 (24%)	265 (27%)
Ex since cancer diagnosis	83 (4.1%)	46 (4.4%)	37 (3.8%)
Current	282 (14%)	168 (16%)	114 (12%)
Unknown	22	10	12
Ethnicity			
White (European or American European)	1933 (94%)	983 (93%)	950 (96%)
Other	117 (5.7%)	74 (7.0%)	43 (4.3%)
Unknown	6	5	1
BMI^b^ (kg/m^2^) [Median]	25.5 (22.6, 29.3)	24.7 (21.9, 28.8)	26.2 (23.2, 30.0)
Unknown	20	8	12
Number nodes involved			
0	1474 (79%)	689 (74%)	785 (83%)
1	228 (12%)	127 (14%)	101 (11%)
2	75 (4.0%)	52 (5.6%)	23 (2.4%)
3+	93 (5.0%)	58 (6.3%)	35 (3.7%)
Unknown	186	136	50
Oestrogen receptor status			
Positive	1666 (86%)	796 (84%)	870 (88%)
Negative	266 (14%)	152 (16%)	114 (12%)
Unknown	124	114	10
HER2 status			
Positive	206 (12%)	117 (13%)	89 (9.8%)
Negative	1580 (88%)	759 (87%)	821 (90%)
Unknown	270	186	84
Radiotherapy IMRT^c^			
No	1035 (50%)	723 (68%)	312 (31%)
Yes	1018 (50%)	336 (32%)	682 (69%)
Unknown	3	3	0
Radiotherapy treatment position			
Prone	299 (15%)	49 (4.6%)	250 (25%)
Supine	1756 (85%)	1012 (95%)	744 (75%)
Unknown	1	1	0
Radiotherapy boost			
No	662 (32%)	203 (19%)	459 (46%)
Yes	1394 (68%)	859 (81%)	535 (54%)
Radiotherapy boost sequence			
Sequential including IORT^d^ boost	1137 (82%)	680 (79%)	457 (85%)
Simultaneous integrated	217 (16%)	139 (16%)	78 (15%)
Simultaneous integrated and sequential^e^	40 (2.9%)	40 (4.7)	0 (0%)
Radiotherapy breast dose Gy [Median]	50.0 (40.1, 50.0)	50.0 (50.0, 50.0)	40.1 (40.1, 40.5)
Radiotherapy breast fractions	25 (15, 25)	25 (25, 26)	15 (15, 15)
Breast surgery type			
Segmentectomy/Quadrantectomy	1117 (55%)	830 (80%)	287 (29%)
Wide local excision	918 (45%)	211 (20%)	707 (71%)
Unknown	21	21	0
Tumour side of primary			
Left	1048 (51%)	562 (54%)	486 (49%)
Right	996 (49%)	488 (46%)	508 (51%)
Unknown	12	12	0
Pathological tumour size mm [Median]	14 (9, 20)	14 (9, 20)	14 (9, 20)
Pathologic UICC T-stage			
Tis	252 (13%)	182 (18%)	70 (7.1%)
T1	1361 (68%)	628 (62%)	733 (74%)
T2	333 (17%)	163 (16%)	170 (17%)
T3	8 (0.4%)	6 (0.6%)	2 (0.2%)
T4	5 (0.3%)	3 (0.3%)	2 (0.2%)
TX	36 (1.8%)	27 (2.7%)	9 (0.9%)
Unknown	61	53	8
Neoadjuvant UICC T-stage			
Tis	3 (1.4%)	2 (1.3%)	1 (1.8%)
T1	63 (30%)	45 (29%)	18 (32%)
T2	130 (62%)	97 (63%)	33 (59%)
T3	9 (4.3%)	7 (4.5%)	2 (3.6%)
TX	5 (2.4%)	3 (1.9%)	2 (3.6%)
Chemotherapy adjuvant			
No	1585 (78%)	785 (75%)	800 (80%)
Yes	460 (22%)	266 (25%)	194 (20%)
Unknown	11	11	0
Chemotherapy neoadjuvant			
No	1853 (91%)	900 (86%)	953 (96%)
Yes	192 (9.4%)	151 (14%)	41 (4.1%)
Unknown	11	11	0
Tamoxifen			
No	801 (49%)	397 (51%)	404 (47%)
Yes	828 (51%)	378 (49%)	450 (53%)
Unknown	427	287	140
Aromatase Inhibitor			
No	817 (50%)	387 (50%)	430 (51%)
Yes	808 (50%)	387 (50%)	421 (49%)
Unknown	431	288	143
Radiotherapy supraclavicular fossa			
0 = No	1777 (87%)	842 (80%)	935 (94%)
1 = Yes	264 (13%)	208 (20%)	56 (5.7%)
Unknown	15	12	3

Percentages are calculated using the number of patients with available data as the denominator and therefore sum to 100% across the reported categories. Patients with unknown or missing data are reported as absolute numbers only.

a n (%); Median (Q1, Q3).

b BMI: Body mass index.

c IMRT: Intensity modulated radiation therapy.

d IORT: Intraoperative radiation therapy.

e WBI = whole-breast irradiation, 28 × 1.8 Gy to 50.4 Gy; SIB = simultaneous integrated boost, 28 × 0.2 Gy to 56 Gy; SEB = sequential external boost, 2 × 2 Gy to a maximum total dose of 60 Gy.

CTCAE outcome data are presented in Table 2 and Fig. 2 (Supplementary Fig. 1 for relative values). For the combined 5–8-year category, the maximum grade recorded for each patient across this period was used. The side-effects observed in patients receiving a fraction dose of ≤2.2 Gy are described separately with those in patients receiving >2.2 Gy per fraction; detailed dose-fractionation schedules and their frequencies in this cohort are reported elsewhere [4]. Patient-reported outcomes (PROs) were summarised as scale scores according to the EORTC scoring manual. For each scale, the median, mean, and standard deviation were calculated. Table 3 presents the EORTC QLQ-C30 questionnaire, and Table 4 presents the EORTC QLQ-BR23 questionnaire.

**Table 2:** Endpoints – CTCAE symptoms by follow-up timepoints, stratified by fraction size (≤ 2.2 Gy vs. > 2.2 Gy) in absolute and relative frequencies.

Fraction size	Characteristic	Baseline	PostRT	1 yr	2 yr	3 yr	4 yr	5–8 yr
		N = 1062^1^	*N* = 1058^1^	*N* = 997^1^	*N* = 953^1^	*N* = 370^1^	*N* = 168^1^	*N* = 590^1^
≤ 2.2 Gy per fraction	Atrophy							
	0 = None	729 (69%)	677 (65%)	631 (64%)	536 (56%)	215 (59%)	114 (69%)	287 (52%)
	1 = Minimal asymmetry; minimal atrophy	273 (26%)	249 (24%)	258 (26%)	309 (33%)	117 (32%)	36 (22%)	187 (34%)
	2 = Moderate asymmetry; moderate atrophy	45 (4.2%)	106 (10%)	93 (9.4%)	99 (10%)	31 (8.5%)	13 (7.9%)	66 (12%)
	3 = Asymmetry >1/3 of breast volume; severe atrophy	13 (1.2%)	3 (0.3%)	5 (0.5%)	5 (0.5%)	2 (0.5%)	2 (1.2%)	9 (1.6%)
	Unknown	2	23	10	4	5	3	41
	Nipple retraction							
	0 = None	949 (90%)	932 (91%)	895 (91%)	844 (89%)	332 (91%)	150 (91%)	506 (91%)
	1 = Asymptomatic; asymmetry with slight retraction and/or thickening of the nipple areolar complex	95 (9.0%)	87 (8.5%)	73 (7.4%)	88 (9.3%)	30 (8.2%)	12 (7.3%)	42 (7.6%)
	2 = Symptomatic; asymmetry of nipple areolar complex with moderate retraction and/or thickening of the nipple areolar complex	6 (0.6%)	6 (0.6%)	14 (1.4%)	13 (1.4%)	3 (0.8%)	3 (1.8%)	6 (1.1%)
	Unknown	12	33	15	8	5	3	36
	Oedema							
	0 = None	925 (87%)	654 (63%)	767 (77%)	834 (88%)	330 (90%)	151 (92%)	521 (94%)
	1 = Swelling or obscuration of anatomic architecture on close inspection	122 (12%)	357 (34%)	185 (19%)	101 (11%)	33 (9.0%)	13 (7.9%)	30 (5.4%)
	2 = Readily apparent obscuration of anatomic architecture; obliteration of skin folds; readily apparent deviation from normal anatomic contour; limiting instrumental ADL	11 (1.0%)	31 (3.0%)	38 (3.8%)	18 (1.9%)	2 (0.5%)	1 (0.6%)	3 (0.5%)
	3 = Gross deviation from normal anatomic contour; limiting self-care ADL	2 (0.2%)	2 (0.2%)	1 (0.1%)	0 (0%)	1 (0.3%)	0 (0%)	0 (0%)
	Unknown	2	14	6	0	4	3	36
	Skin ulceration							
	0 = None	1054 (99%)	909 (87%)	987 (100%)	947 (99%)	366 (100%)	163 (99%)	553 (100%)
	1 = Combined area of ulcers <1 cm no blanchable erythema of intact skin with associated warmth or edema	6 (0.6%)	84 (8.0%)	3 (0.3%)	6 (0.6%)	0 (0%)	1 (0.6%)	1 (0.2%)
	2 = Combined area of ulcers 1-2 cm; partial thickness skin loss involving skin or subcutaneous fat	0 (0%)	39 (3.7%)	1 (0.1%)	0 (0%)	0 (0%)	0 (0%)	0 (0%)
	3 = Combined area of ulcers >2 cm; full thickness skin loss involving damage to or necrosis of subcutaneous tissue that may extend down to fascia	0 (0%)	12 (1.1%)	0 (0%)	0 (0%)	0 (0%)	1 (0.6%)	0 (0%)
	Unknown	2	14	6	0	4	3	36
	Telangiectasia tumour bed							
	0 = None	1045 (99%)	1036 (99%)	966 (98%)	908 (95%)	348 (95%)	151 (92%)	502 (90%)
	1 = Telangiectasia covering <10% of the treated breast	12 (1.1%)	6 (0.6%)	19 (1.9%)	43 (4.5%)	16 (4.4%)	13 (7.9%)	51 (9.2%)
	2 = Telangiectasia covering >10% of the treated breast; associated with psychosocial impact	2 (0.2%)	1 (<0.1%)	3 (0.3%)	2 (0.2%)	2 (0.5%)	1 (0.6%)	2 (0.4%)
	Unknown	3	15	9	0	4	3	35
	Telangiectasia outside tumour bed							
	0 = None	1045 (99%)	1035 (99%)	956 (97%)	925 (97%)	355 (97%)	153 (93%)	512 (92%)
	1 = Telangiectasia covering <10% of the treated breast	11 (1.0%)	8 (0.8%)	27 (2.7%)	26 (2.7%)	11 (3.0%)	12 (7.3%)	36 (6.5%)
	2 = Telangiectasia covering >10% of the treated breast; associated with psychosocial impact	2 (0.2%)	0 (0%)	5 (0.5%)	2 (0.2%)	0 (0%)	0 (0%)	6 (1.1%)
	Unknown	4	15	9	0	4	3	36
	Skin induration tumour bed							
	0 = None	699 (66%)	688 (66%)	584 (59%)	605 (63%)	246 (67%)	115 (70%)	293 (53%)
	1 = Mild induration; able to move skin parallel to plane (sliding) and perpendicular to skin (pinching up)	294 (28%)	289 (28%)	320 (32%)	283 (30%)	96 (26%)	47 (28%)	206 (37%)
	2 = Moderate induration; able to slide skin; unable to pinch skin; limiting instrumental activities for daily living (ADL)	56 (5.3%)	57 (5.5%)	74 (7.5%)	57 (6.0%)	23 (6.3%)	3 (1.8%)	49 (8.9%)
	3 = Severe induration; unable to slide or pinch skin; affecting activities for daily living; limiting self-care ADL	10 (0.9%)	3 (0.3%)	11 (1.1%)	8 (0.8%)	0 (0%)	0 (0%)	5 (0.9%)
	Unknown	3	21	8	0	5	3	37
	Skin induration outside tumour bed							
	0 = None	974 (92%)	970 (94%)	846 (86%)	836 (88%)	320 (87%)	153 (93%)	458 (83%)
	1 = Mild induration; able to move skin parallel to plane (sliding) and perpendicular to skin (pinching up)	71 (6.7%)	57 (5.5%)	116 (12%)	102 (11%)	41 (11%)	9 (5.5%)	85 (15%)
	2 = Moderate induration; able to slide skin; unable to pinch skin; limiting instrumental activities for daily living (ADL)	12 (1.1%)	8 (0.8%)	23 (2.3%)	12 (1.3%)	4 (1.1%)	3 (1.8%)	11 (2.0%)
	3 = Severe induration; unable to slide or pinch skin; affecting activities for daily living; limiting self-care ADL	2 (0.2%)	2 (0.2%)	4 (0.4%)	3 (0.3%)	1 (0.3%)	0 (0%)	1 (0.2%)
	Unknown	3	21	8	0	4	3	35
	Erythema							
	0 = None	975 (92%)	101 (9.6%)	923 (93%)	922 (97%)	361 (99%)	163 (99%)	555 (100%)
	1 = Faint erythema or dry desquamation	78 (7.4%)	617 (59%)	64 (6.5%)	29 (3.0%)	5 (1.4%)	2 (1.2%)	0 (0%)
	2 = Moderate to brisk erythema; patchy moist desquamation mostly confined to skin folds and creases; moderate oedema	6 (0.6%)	305 (29%)	2 (0.2%)	1 (0.1%)	0 (0%)	0 (0%)	1 (0.2%)
	3 = Moist desquamation in areas other than skin folds and creases; bleeding induced by minor trauma or abrasion	0 (0%)	24 (2.3%)	0 (0%)	0 (0%)	0 (0%)	0 (0%)	1 (0.2%)
	Unknown	3	11	8	1	4	3	33
	Arm lymphoedema							
	0 = None	1029 (97%)	1013 (97%)	945 (96%)	903 (95%)	351 (96%)	157 (95%)	532 (95%)
	1 = 5–10% inter limb discrepancy in volume or circumference at point of greatest visible difference; swelling or obscuration of anatomic architecture on close inspection	28 (2.6%)	29 (2.8%)	37 (3.7%)	43 (4.5%)	10 (2.7%)	5 (3.0%)	25 (4.5%)
	2 ≥10–30% inter limb discrepancy in volume or circumference at point of greatest visible difference; Readily apparent obscuration of anatomic architecture; obliteration of skin folds; readily apparent deviation from normal anatomic contour; limiting instrumental activities of daily living (ADL)	3 (0.3%)	3 (0.3%)	6 (0.6%)	7 (0.7%)	4 (1.1%)	3 (1.8%)	1 (0.2%)
	3 ≥30% inter-limb discrepancy in volume; gross deviation from normal anatomic contour; limiting self-care ADL	0 (0%)	0 (0%)	0 (0%)	0 (0%)	1 (0.3%)	0 (0%)	1 (0.2%)
	Unknown	2	13	9	0	4	3	31
	Skin hyperpigmentation							
	0 = None	1021 (96%)	733 (70%)	598 (61%)	745 (78%)	312 (85%)	151 (92%)	500 (90%)
	1 = Hyperpigmentation or depigmentation covering <10% body surface area (BSA); no psychosocial impact	38 (3.6%)	294 (28%)	381 (39%)	206 (22%)	54 (15%)	14 (8.5%)	53 (9.6%)
	2 = Hyperpigmentation or depigmentation covering >10% BSA; associated with psychosocial impact	1 (<0.1%)	16 (1.5%)	9 (0.9%)	2 (0.2%)	0 (0%)	0 (0%)	0 (0%)
	Unknown	2	15	9	0	4	3	37
	Pneumonitis							
	0 = None	1042 (100%)	1028 (100%)	981 (99%)	951 (100%)	366 (100%)	165 (100%)	553 (100%)
	1 = Asymptomatic; clinical or diagnostic observations only; intervention not indicated	1 (<0.1%)	2 (0.2%)	10 (1.0%)	2 (0.2%)	0 (0%)	0 (0%)	0 (0%)
	2 = Symptomatic; medical intervention indicated; limiting instrumental activities of daily living (ADL)	1 (<0.1%)	2 (0.2%)	3 (0.3%)	0 (0%)	0 (0%)	0 (0%)	1 (0.2%)
	3 = Severe symptoms; limiting self-care ADL; oxygen indicated	1 (<0.1%)	0 (0%)	0 (0%)	0 (0%)	0 (0%)	0 (0%)	0 (0%)
	Unknown	17	26	3	0	4	3	36
	Pain							
	0 = None	705 (67%)	576 (55%)	643 (65%)	667 (70%)	281 (76%)	129 (82%)	392 (74%)
	1 = Mild	294 (28%)	354 (34%)	268 (27%)	207 (22%)	63 (17%)	26 (16%)	81 (15%)
	2 = Moderate; limiting usual activities	47 (4.4%)	86 (8.2%)	55 (5.5%)	57 (6.0%)	17 (4.6%)	3 (1.9%)	42 (7.9%)
	3 = Severe - stopping activities	14 (1.3%)	29 (2.8%)	25 (2.5%)	21 (2.2%)	7 (1.9%)	0 (0%)	17 (3.2%)
	Unknown	2	13	6	1	2	10	58
	Pain medication							
	1 = Yes	44 (12%)	74 (16%)	47 (14%)	25 (8.8%)	8 (9.2%)	0 (0%)	17 (12%)
	0 = No	311 (88%)	397 (84%)	301 (86%)	260 (91%)	79 (91%)	29 (100%)	121 (88%)
	Unknown	707	587	649	668	283	139	452
	Swollen arm							
	1 = Yes	62 (5.8%)	51 (4.9%)	78 (7.9%)	80 (8.4%)	25 (6.8%)	5 (3.4%)	54 (10%)
	0 = No	998 (94%)	996 (95%)	906 (92%)	867 (92%)	343 (93%)	144 (97%)	486 (90%)
	Unknown	2	11	13	6	2	19	50


Fraction size	Characteristic	Baseline	PostRT	1 yr	2 yr	3 yr	4 yr	5–8 yr
		*N* = 994^1^	*N* = 992^1^	*N* = 876^1^	*N* = 806^1^	*N* = 293^1^	*N* = 265^1^	*N* = 389^1^
> 2.2 Gy per fraction	Atrophy							
	0 = None	757 (76%)	675 (71%)	518 (61%)	392 (49%)	121 (48%)	109 (57%)	129 (45%)
	1 = Minimal asymmetry; minimal atrophy	203 (20%)	241 (25%)	257 (30%)	286 (36%)	89 (35%)	60 (31%)	116 (41%)
	2 = Moderate asymmetry; moderate atrophy	33 (3.3%)	39 (4.1%)	69 (8.1%)	102 (13%)	35 (14%)	21 (11%)	36 (13%)
	3 = Asymmetry >1/3 of breast volume; severe atrophy	1 (0.1%)	1 (0.1%)	7 (0.8%)	19 (2.4%)	7 (2.8%)	2 (1.0%)	5 (1.7%)
	Unknown	0	36	25	7	41	73	103
	Nipple retraction							
	0 = None	872 (90%)	821 (87%)	733 (87%)	654 (83%)	215 (86%)	171 (90%)	243 (85%)
	1 = Asymptomatic; asymmetry with slight retraction and/or thickening of the nipple areolar complex	91 (9.4%)	109 (12%)	104 (12%)	126 (16%)	33 (13%)	16 (8.5%)	30 (11%)
	2 = Symptomatic; asymmetry of nipple areolar complex with moderate retraction and/or thickening of the nipple areolar complex	10 (1.0%)	9 (1.0%)	9 (1.1%)	6 (0.8%)	1 (0.4%)	2 (1.1%)	12 (4.2%)
	Unknown	21	53	30	20	44	76	104
	Oedema							
	0 = None	901 (91%)	569 (59%)	682 (79%)	695 (87%)	221 (88%)	172 (90%)	267 (94%)
	1 = Swelling or obscuration of anatomic architecture on close inspection	90 (9.1%)	394 (41%)	157 (18%)	94 (12%)	25 (9.9%)	19 (9.9%)	18 (6.3%)
	2 = Readily apparent obscuration of anatomic architecture; obliteration of skin folds; readily apparent deviation from normal anatomic contour; limiting instrumental ADL	3 (0.3%)	8 (0.8%)	21 (2.4%)	12 (1.5%)	6 (2.4%)	0 (0%)	0 (0%)
	3 = Gross deviation from normal anatomic contour; limiting self-care ADL	0 (0%)	0 (0%)	0 (0%)	0 (0%)	0 (0%)	0 (0%)	0 (0%)
	Unknown	0	21	16	5	41	74	104
	Skin ulceration							
	0 = None	989 (100%)	922 (95%)	851 (99%)	795 (99%)	252 (100%)	191 (100%)	285 (100%)
	1 = Combined area of ulcers <1 cm no blanchable erythema of intact skin with associated warmth or edema	3 (0.3%)	39 (4.0%)	7 (0.8%)	5 (0.6%)	0 (0%)	0 (0%)	0 (0%)
	2 = Combined area of ulcers 1-2 cm; partial thickness skin loss involving skin or subcutaneous fat	0 (0%)	7 (0.7%)	0 (0%)	1 (0.1%)	0 (0%)	0 (0%)	0 (0%)
	3 = Combined area of ulcers >2 cm; full thickness skin loss involving damage to or necrosis of subcutaneous tissue that may extend down to fascia	0 (0%)	1 (0.1%)	0 (0%)	0 (0%)	0 (0%)	0 (0%)	0 (0%)
	Unknown	2	23	18	5	41	74	104
	Telangiectasia tumour bed							
	0 = None	992 (100%)	969 (100%)	841 (98%)	764 (95%)	244 (97%)	175 (92%)	263 (91%)
	1 = Telangiectasia covering <10% of the treated breast	2 (0.2%)	3 (0.3%)	13 (1.5%)	34 (4.2%)	8 (3.2%)	15 (7.9%)	23 (7.9%)
	2 = Telangiectasia covering >10% of the treated breast; associated with psychosocial impact	0 (0%)	0 (0%)	0 (0%)	3 (0.4%)	0 (0%)	1 (0.5%)	4 (1.4%)
	Unknown	0	20	22	5	41	74	99
	Telangiectasia outside tumour bed							
	0 = None	985 (99%)	966 (99%)	819 (96%)	754 (94%)	240 (95%)	177 (93%)	264 (92%)
	1 = Telangiectasia covering <10% of the treated breast	9 (0.9%)	5 (0.5%)	34 (4.0%)	44 (5.5%)	12 (4.8%)	12 (6.3%)	22 (7.7%)
	2 = Telangiectasia covering >10% of the treated breast; associated with psychosocial impact	0 (0%)	0 (0%)	1 (0.1%)	3 (0.4%)	0 (0%)	2 (1.0%)	1 (0.3%)
	Unknown	0	21	22	5	41	74	102
	Skin induration tumour bed							
	0 = None	558 (56%)	540 (56%)	434 (51%)	463 (58%)	128 (51%)	86 (45%)	135 (47%)
	1 = Mild induration; able to move skin parallel to plane (sliding) and perpendicular to skin (pinching up)	406 (41%)	396 (41%)	368 (43%)	291 (36%)	109 (43%)	90 (47%)	126 (44%)
	2 = Moderate induration; able to slide skin; unable to pinch skin; limiting instrumental activities for daily living (ADL)	28 (2.8%)	27 (2.8%)	50 (5.9%)	48 (6.0%)	14 (5.6%)	13 (6.8%)	23 (8.1%)
	3 = Severe induration; unable to slide or pinch skin; affecting activities for daily living; limiting self care ADL	0 (0%)	2 (0.2%)	1 (0.1%)	0 (0%)	1 (0.4%)	2 (1.0%)	1 (0.4%)
	Unknown	2	27	23	4	41	74	104
	Skin induration outside tumour bed							
	0 = None	944 (95%)	913 (95%)	709 (83%)	670 (84%)	187 (74%)	151 (79%)	242 (84%)
	1 = Mild induration; able to move skin parallel to plane (sliding) and perpendicular to skin (pinching up)	41 (4.1%)	47 (4.9%)	138 (16%)	120 (15%)	61 (24%)	34 (18%)	35 (12%)
	2 = Moderate induration; able to slide skin; unable to pinch skin; limiting instrumental activities for daily living (ADL)	6 (0.6%)	4 (0.4%)	6 (0.7%)	11 (1.4%)	4 (1.6%)	6 (3.1%)	10 (3.5%)
	3 = Severe induration; unable to slide or pinch skin; affecting activities for daily living; limiting self-care ADL	1 (0.1%)	1 (0.1%)	1 (0.1%)	0 (0%)	0 (0%)	0 (0%)	0 (0%)
	Unknown	2	27	22	5	41	74	102
	Erythema							
	0 = None	950 (96%)	156 (16%)	808 (95%)	770 (96%)	248 (98%)	187 (98%)	311 (99%)
	1 = Faint erythema or dry desquamation	42 (4.2%)	669 (68%)	45 (5.3%)	30 (3.8%)	4 (1.6%)	4 (2.1%)	2 (0.6%)
	2 = Moderate to brisk erythema; patchy moist desquamation mostly confined to skin folds and creases; moderate oedema	2 (0.2%)	157 (16%)	0 (0%)	0 (0%)	0 (0%)	0 (0%)	0 (0%)
	3 = Moist desquamation in areas other than skin folds and creases; bleeding induced by minor trauma or abrasion	0 (0%)	4 (0.4%)	0 (0%)	0 (0%)	0 (0%)	0 (0%)	0 (0%)
	Unknown	0	6	23	6	41	74	76
	Arm lymphoedema							
	0 = None	980 (99%)	947 (97%)	837 (98%)	783 (98%)	245 (97%)	184 (96%)	318 (98%)
	1 = 5–10% inter limb discrepancy in volume or circumference at point of greatest visible difference; swelling or obscuration of anatomic architecture on close inspection	14 (1.4%)	24 (2.5%)	15 (1.8%)	15 (1.9%)	4 (1.6%)	7 (3.7%)	8 (2.5%)
	2 ≥10–30% inter limb discrepancy in volume or circumference at point of greatest visible difference; Readily apparent obscuration of anatomic architecture; obliteration of skin folds; readily apparent deviation from normal anatomic contour; limiting instrumental activities of daily living (ADL)	0 (0%)	2 (0.2%)	2 (0.2%)	2 (0.3%)	3 (1.2%)	0 (0%)	0 (0%)
	3 ≥30% inter-limb discrepancy in volume; gross deviation from normal anatomic contour; limiting self-care ADL	0 (0%)	0 (0%)	0 (0%)	0 (0%)	0 (0%)	0 (0%)	0 (0%)
	Unknown	0	19	22	6	41	74	63
	Skin hyperpigmentation							
	0 = None	985 (99%)	880 (91%)	622 (73%)	685 (86%)	229 (91%)	178 (93%)	265 (92%)
	1 = Hyperpigmentation or depigmentation covering <10% body surface area (BSA); no psychosocial impact	8 (0.8%)	77 (8.0%)	226 (27%)	113 (14%)	23 (9.1%)	13 (6.8%)	22 (7.6%)
	2 = Hyperpigmentation or depigmentation covering >10% BSA; associated with psychosocial impact	1 (0.1%)	11 (1.1%)	4 (0.5%)	2 (0.3%)	0 (0%)	0 (0%)	2 (0.7%)
	Unknown	0	24	24	6	41	74	100
	Pneumonitis							
	0 = None	991 (100%)	969 (99%)	853 (99%)	796 (99%)	252 (100%)	190 (99%)	291 (100%)
	1 = Asymptomatic; clinical or diagnostic observations only; intervention not indicated	2 (0.2%)	4 (0.4%)	4 (0.5%)	2 (0.2%)	0 (0%)	0 (0%)	0 (0%)
	2 = Symptomatic; medical intervention indicated; limiting instrumental activities of daily living (ADL)	1 (0.1%)	1 (0.1%)	3 (0.3%)	3 (0.4%)	0 (0%)	1 (0.5%)	0 (0%)
	3 = Severe symptoms; limiting self-care ADL; oxygen indicated	0 (0%)	0 (0%)	0 (0%)	0 (0%)	0 (0%)	0 (0%)	0 (0%)
	Unknown	0	18	16	5	41	74	98
	Pain							
	0 = None	710 (72%)	497 (51%)	580 (67%)	605 (75%)	217 (75%)	192 (74%)	269 (76%)
	1 = Mild	265 (27%)	430 (44%)	266 (31%)	178 (22%)	55 (19%)	49 (19%)	70 (20%)
	2 = Moderate; limiting usual activities	18 (1.8%)	41 (4.2%)	21 (2.4%)	21 (2.6%)	18 (6.2%)	17 (6.6%)	15 (4.2%)
	3 = Severe - stopping activities	0 (0%)	1 (0.1%)	3 (0.3%)	1 (0.1%)	0 (0%)	1 (0.4%)	0 (0%)
	Unknown	1	23	6	1	3	6	35
	Pain medication							
	1 = Yes	36 (13%)	50 (11%)	20 (6.9%)	15 (7.5%)	5 (6.8%)	6 (9.0%)	8 (9.4%)
	0 = No	247 (87%)	422 (89%)	270 (93%)	185 (93%)	68 (93%)	61 (91%)	77 (91%)
	Unknown	711	520	586	606	220	198	304
	Swollen arm							
	1 = Yes	52 (5.2%)	62 (6.4%)	44 (5.1%)	33 (4.1%)	11 (3.8%)	19 (7.5%)	25 (6.9%)
	0 = No	941 (95%)	907 (94%)	822 (95%)	770 (96%)	279 (96%)	233 (92%)	336 (93%)
	Unknown	1	23	10	3	3	13	28

1 n (%); Median (Q1, Q3) Percentages are calculated using the number of patients with available data as the denominator and therefore sum to 100% across the reported grades. Patients with unknown or missing data are reported as absolute numbers only. For the combined 5–8-year category, the maximum grade recorded for each patient within this period is shown.

**Fig. 2:**
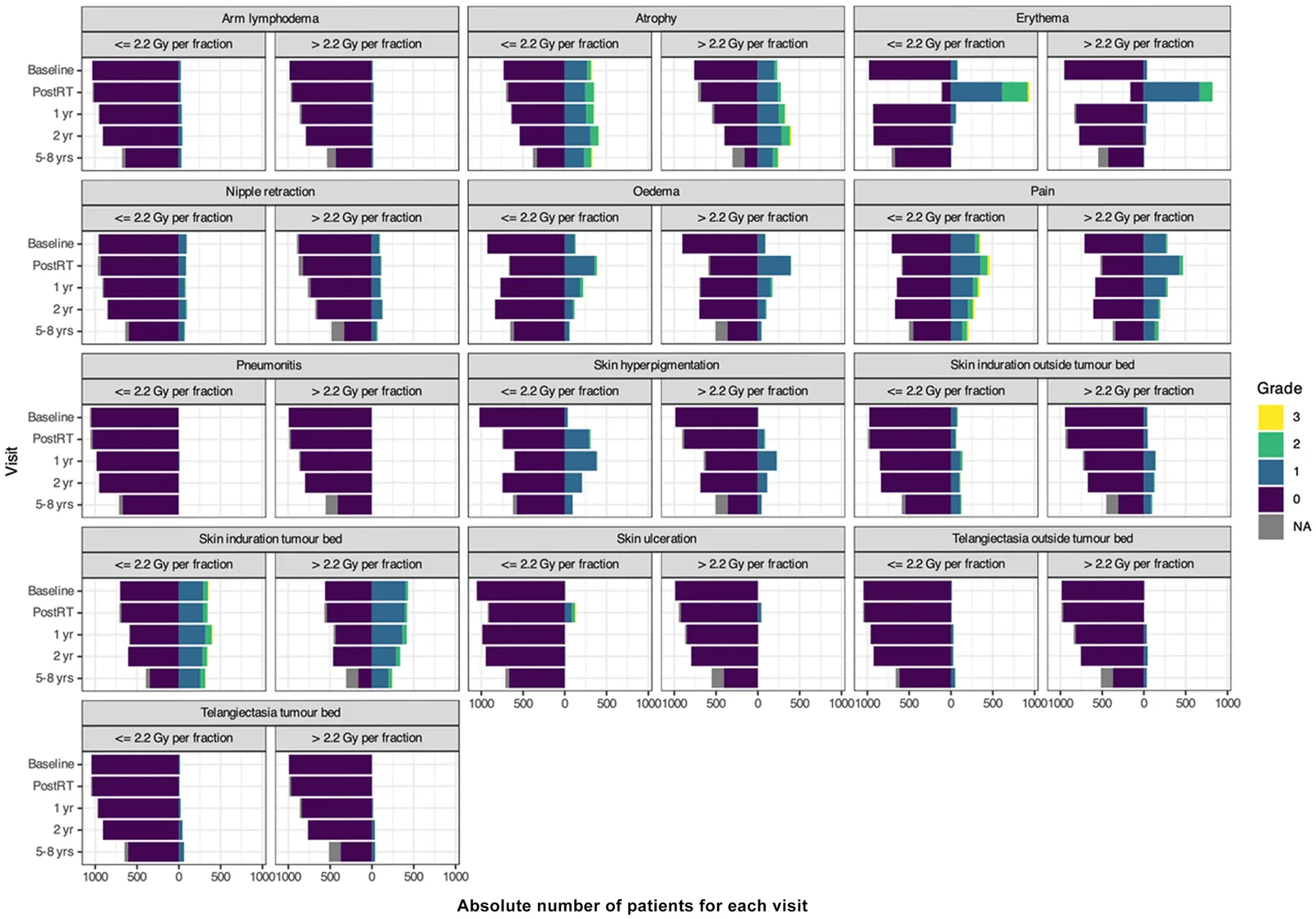
Endpoints – CTCAE symptoms by follow-up timepoints, stratified by fraction size (≤ 2.2 Gy vs. > 2.2 Gy) in absolute frequencies.

**Table 3:** Patient reported outcomes – EORTC QLQ C30 scale scores.

Group	Characteristic	Baseline *N* = 2025	PostRT *N* = 1882	1 yr *N* = 1745	2 yr *N* = 1623	3 yr *N* = 587	4 yr *N* = 468	5 yr *N* = 591	6 yr *N* = 211	7 yr *N* = 201	8 yr *N* = 34
Quality of life (QL)	score										
	N Non-missing	1989	1837	1719	1560	568	461	578	207	199	34
	Mean (SD)	68 (20)	66 (20)	73 (19)	73 (19)	73 (20)	73 (19)	74 (19)	75 (19)	75 (18)	79 (19)
	Median (Q1, Q3)	67 (50, 83)	67 (50, 83)	75 (67, 83)	75 (67, 83)	75 (67, 83)	75 (67, 83)	83 (67, 83)	83 (67, 92)	83 (67, 83)	83 (67, 92)
Physical function (PF)	score										
	N Non-missing	1999	1854	1729	1576	574	465	586	209	200	34
	Mean (SD)	85 (17)	84 (17)	86 (17)	87 (16)	87 (16)	87 (16)	88 (16)	89 (14)	90 (14)	91 (17)
	Median (Q1, Q3)	93 (80, 100)	87 (73, 100)	93 (80, 100)	93 (80, 100)	93 (80, 100)	93 (80, 100)	93 (80, 100)	93 (80, 100)	93 (87, 100)	100 (93, 100)
Role function (RF)	score										
	N Non-missing	1998	1850	1723	1572	570	460	568	209	199	34
	Mean (SD)	78 (26)	74 (26)	83 (23)	84 (23)	84 (24)	86 (21)	86 (22)	86 (21)	89 (18)	91 (20)
	Median (Q1, Q3)	83 (67, 100)	83 (67, 100)	100 (67, 100)	100 (67, 100)	100 (67, 100)	100 (67, 100)	100 (67, 100)	100 (67, 100)	100 (83, 100)	100 (83, 100)
Emotional function (EF)	score										
	N Non-missing	1995	1849	1718	1568	572	462	571	207	199	34
	Mean (SD)	73 (23)	74 (23)	77 (23)	77 (23)	78 (23)	80 (21)	79 (23)	79 (22)	80 (22)	81 (23)
	Median (Q1, Q3)	75 (67, 92)	75 (67, 92)	83 (67, 100)	83 (67, 100)	83 (67, 100)	83 (67, 100)	83 (67, 100)	83 (67, 100)	83 (67, 100)	83 (75, 100)
Cognitive function (CF)	score										
	N Non-missing	1993	1850	1721	1569	571	462	571	207	200	34
	Mean (SD)	84 (21)	81 (23)	81 (23)	81 (22)	80 (23)	83 (22)	82 (22)	82 (24)	83 (22)	83 (25)
	Median (Q1, Q3)	83 (67, 100)	83 (67, 100)	83 (67, 100)	83 (67, 100)	83 (67, 100)	83 (67, 100)	83 (67, 100)	83 (67, 100)	83 (67, 100)	83 (83, 100)
Social function (SF)	score										
	N Non-missing	1993	1850	1721	1568	573	462	570	206	199	34
	Mean (SD)	80 (25)	79 (24)	86 (22)	88 (21)	89 (19)	88 (21)	90 (20)	89 (19)	91 (19)	90 (23)
	Median (Q1, Q3)	83 (67, 100)	83 (67, 100)	100 (83, 100)	100 (83, 100)	100 (83, 100)	100 (83, 100)	100 (83, 100)	100 (83, 100)	100 (100,100)	100 (100, 100)
Fatigue (FA)	score										
	N Non-missing	1993	1847	1720	1566	571	460	568	206	199	34
	Mean (SD)	30 (24)	38 (25)	28 (24)	28 (23)	28 (25)	24 (23)	24 (23)	22 (23)	21 (21)	20 (24)
	Median (Q1, Q3)	33 (11, 44)	33 (22, 56)	22 (11, 44)	22 (11,33)	22 (11, 44)	22 (0,33)	22 (0, 33)	22 (0, 33)	22 (0, 33)	11 (0, 33)
Nausea/Vomiting (NV)	score										
	N Non-missing	1992	1849	1720	1570	571	461	572	206	199	34
	Mean (SD)	3.5 (10.4)	4.9 (11.3)	3.3 (9.9)	3.3 (10.0)	3.1 (9.1)	3.0 (9.3)	3.0 (9.4)	2.6 (7.3)	1.9 (6.7)	2.9 (10.4)
	Median (Q1, Q3)	0.0 (0.0, 0.0)	0.0 (0.0, 0.0)	0.0 (0.0, 0.0)	0.0 (0.0, 0.0)	0.0 (0.0, 0.0)	0.0 (0.0, 0.0)	0.0 (0.0, 0.0)	0.0 (0.0, 0.0)	0.0 (0.0, 0.0)	0.0 (0.0, 0.0)
Pain (PA)	score										
	N Non-missing	1995	1850	1721	1570	569	462	571	207	200	34
	Mean (SD)	20 (23)	25 (24)	20 (24)	19 (24)	18 (23)	18 (22)	18 (23)	17 (23)	14 (22)	14 (22)
	Median (Q1, Q3)	17 (0, 33)	17 (0, 33)	17 (0, 33)	17 (0, 33)	17 (0, 33)	17 (0, 33)	17 (0, 33)	17 (0, 33)	0 (0, 17)	0 (0, 17)
Dyspnea (DY)	score										
	N Non-missing	1992	1843	1700	1560	562	453	557	207	199	33
	Mean (SD)	14 (23)	17 (24)	17 (25)	17 (25)	16 (24)	14 (23)	15 (22)	14 (22)	14 (21)	16 (25)
	Median (Q1, Q3)	0 (0, 33)	0 (0, 33)	0 (0, 33)	0 (0, 33)	0 (0, 33)	0 (0, 33)	0 (0, 33)	0 (0, 33)	0 (0, 33)	0 (0, 33)
Insomnia (SL)	score										
	N Non-missing	1979	1839	1709	1550	567	459	565	207	197	34
	Mean (SD)	33 (31)	34 (32)	33 (33)	33 (32)	33 (31)	31 (32)	30 (31)	32 (30)	27 (29)	29 (32)
	Median (Q1, Q3)	33 (0, 67)	33 (0, 67)	33 (0, 67)	33 (0, 67)	33 (0, 67)	33 (0, 67)	33 (0, 33)	33 (0, 33)	33 (0, 33)	33 (0, 33)
Appetite loss (AL)	score										
	N Non-missing	1987	1835	1710	1560	565	461	567	206	198	34
	Mean (SD)	8 (19)	11 (21)	6 (15)	6 (16)	5 (15)	6 (16)	5 (15)	4 (12)	5 (16)	7 (20)
	Median (Q1, Q3)	0 (0,0)	0 (0, 33)	0 (0, 0)	0 (0, 0)	0 (0, 0)	0 (0, 0)	0 (0, 0)	0 (0, 0)	0 (0, 0)	0 (0, 0)
Constipation (CO)	score										
	N Non-missing	1986	1844	1711	1565	570	458	569	205	198	34
	Mean (SD)	12 (23)	11 (22)	13 (24)	13 (23)	13 (24)	12 (21)	13 (24)	13 (23)	12 (22)	20 (31)
	Median (Q1, Q3)	0 (0, 0)	0 (0, 0)	0 (0, 33)	0 (0, 33)	0 (0, 33)	0 (0, 33)	0 (0, 33)	0 (0, 33)	0 (0, 33)	0 (0, 33)
Diarrhea (DI)	score										
	N Non-missing	1987	1838	1715	1555	569	462	565	204	197	34
	Mean (SD)	6 (16)	5 (14)	6 (17)	5 (16)	5 (15)	7 (17)	6 (16)	8 (19)	8 (19)	7 (20)
	Median (Q1, Q3)	0 (0, 0)	0 (0, 0)	0 (0, 0)	0 (0, 0)	0 (0, 0)	0 (0, 0)	0 (0, 0)	0 (0, 0)	0 (0, 0)	0 (0, 0)
Financial difficulties (FI)	score										
	N Non-missing	1981	1840	1709	1559	567	457	577	206	199	34
	Mean (SD)	13 (25)	12 (24)	9 (21)	8 (21)	7 (19)	6 (18)	5 (16)	7 (19)	7 (17)	6 (15)
	Median (Q1, Q3)	0 (0, 0)	0 (0, 33)	0 (0, 0)	0 (0, 0)	0 (0, 0)	0 (0, 0)	0 (0, 0)	0 (0, 0)	0 (0, 0)	0 (0, 0)

Values shown as Mean (SD); median (quartile 1, quartile 3). EORTC QLQ-C30, European Organization for Research and Treatment of Cancer Quality of Life Questionnaire Core 30. All scales range 0–100. For global health status and functional scales, higher scores indicate better quality of life/functioning. For symptom scales, higher scores indicate greater symptom burden. A change of ≥10 points is considered clinically relevant.

**Table 4:** Patient reported outcomes – EORTC BR23 scale scores.

Group	Characteristic	Baseline N = 2025	PostRT N = 1882	1 yr N = 1745	2 yr N = 1623	3 yr N = 587	4 yr N = 468	5 yr N = 591	6 yr N = 211	7 yr N = 201	8 yr N = 34
Arm symptoms (AS)	score scaled										
	N Non-missing	1950	1799	1669	1507	546	446	561	203	195	33
	Mean (SD)	16 (19)	15 (19)	16 (20)	15 (19)	15 (20)	14 (20)	12 (18)	11 (18)	9 (16)	9 (15)
	Median (Q1, Q3)	11 (0,22)	11 (0, 22)	11 (0, 22)	11 (0, 22)	0 (0, 22)	0 (0, 22)	0 (0, 22)	0 (0,11)	0 (0, 11)	0 (0, 11)
Body image (BI)	score scaled										
	N Non-missing	1943	1804	1657	1516	555	456	557	201	195	33
	Mean (SD)	80 (25)	79 (25)	85 (22)	86 (21)	87 (21)	86 (21)	87 (22)	87 (21)	86 (20)	86 (25)
	Median (Q1, Q3)	92 (67, 100)	83 (67, 100)	100 (75, 100)	100 (75, 100)	100 (83, 100)	100 (75, 100)	100 (83, 100)	100 (83, 100)	100 (75, 100)	100 (75, 100)
Breast symptoms (BS)	score scaled										
	N Non-missing	1947	1799	1664	1506	552	456	557	205	194	33
	Mean (SD)	19 (17)	36 (22)	19 (19)	16 (16)	14 (16)	14 (17)	14 (15)	13 (16)	11 (14)	14 (15)
	Median (Q1, Q3)	17 (8, 25)	33 (17, 50)	17 (8, 25)	8 (0, 25)	8 (0,21)	8 (0, 21)	8 (0, 17)	8 (0, 17)	8 (0, 17)	8 (0, 17)
Future perspective (FU)	score scaled										
	N Non-missing	1986	1840	1716	1543	556	458	560	205	195	33
	Mean (SD)	54 (30)	56 (30)	62 (29)	63 (29)	64 (29)	62 (29)	68 (27)	64 (29)	66 (29)	66 (31)
	Median (Q1, Q3)	67 (33, 67)	67 (33, 67)	67 (33, 67)	67 (33,100)	67 (33, 100)	67 (33, 67)	67 (67, 100)	67 (67, 67)	67 (67, 100)	67 (67, 100)
Hair loss (HL)	score scaled										
	N Non-missing	434	270	402	382	145	125	149	57	50	10
	Mean (SD)	42 (36)	35 (34)	36 (32)	34 (33)	35 (34)	35 (34)	31 (31)	34 (28)	26 (31)	27 (21)
	Median (Q1, Q3)	33 (0, 67)	33 (0, 67)	33 (0, 67)	33 (0, 67)	33 (0, 67)	33 (0, 67)	33 (0, 33)	33 (0, 33)	33 (0, 33)	33 (0, 33)
Sexual enjoyment (SEE)	score scaled										
	N Non-missing	780	721	815	722	263	219	264	93	100	20
	Mean (SD)	57 (28)	55 (26)	58 (27)	57 (28)	59 (26)	57 (25)	58 (27)	58 (27)	60 (26)	58 (34)
	Median (Q1, Q3)	67 (33, 67)	67 (33, 67)	67 (33, 67)	67 (33, 67)	67 (33, 67)	67 (33, 67)	67 (33, 67)	67 (33, 67)	67 (33, 67)	67 (33, 100)
Sexual function (SEF)	score scaled										
	N Non-missing	1840	1701	1567	1411	514	420	518	195	184	31
	Mean (SD)	20 (23)	21 (23)	26 (25)	25 (24)	26 (25)	26 (24)	25 (24)	24 (24)	25 (22)	28 (22)
	Median (Q1, Q3)	17 (0, 33)	17 (0, 33)	33 (0, 33)	33 (0, 33)	33 (0, 33)	33 (0, 33)	33 (0, 33)	33 (0, 33)	33 (0, 33)	33 (0, 33)
Systemic therapy side-effects (ST)	score scaled										
	N Non-missing	1877	1742	1570	1453	529	439	520	198	192	31
	Mean (SD)	18 (17)	18 (15)	18 (15)	17 (15)	18 (15)	17 (15)	15 (14)	15 (14)	15 (14)	16 (17)
	Median (Q1, Q3)	14 (5, 24)	14 (10, 24)	14 (5, 24)	14 (5, 24)	14 (10, 24)	14 (5, 24)	14 (5, 24)	10 (5, 24)	10 (5, 24)	14 (5, 19)

Values shown as Mean (SD); median (quartile 1, quartile 3). EORTC QLQ-BR23, European Organization for Research and Treatment of Cancer Quality of Life Questionnaire Breast Cancer Module. All scales range 0–100. For functional scales (body image, sexual functioning, sexual enjoyment, future perspective), higher scores indicate better functioning. For symptom scales (systemic therapy side effects, breast symptoms, arm symptoms, upset by hair loss), higher scores indicate greater symptom burden. A change of ≥10 points is considered clinically relevant.

All statistical analyses were conducted using R, version 4.1.1 [11].

## 3 Results

### 3.1 Baseline characteristics and data availability

Data were collected from a total of 2057 women. At baseline, questionnaire completion rates were as follows: 98.4% for the EORTC QLQ-BR23 (2025/2057) and QLQ-C30 (2025/2057), 99.95% for the CTCAE 4.0 (2056/2057), 61.3% (1261/2057) for the GPAQ (not collected in Belgium; planned non-collection), and 12.3% (264/2057) for the HBIS questionnaire (only collected among UK patients; planned non-collection) (Fig. 1).

At the primary follow-up timepoint of 2 years, CTCAE 4.0 data were available from 85% (1759/2057) of patients. At 5 years or beyond, data were available from 979/2057 patients (48%) for CTCAE 4.0, 813/2057 patients (39.5%) for the EORTC QLQ-C30/BR23, 528/2057 patients (25.7%) for the GPAQ, and 45/2057 patients (2.2%) for the HBIS. Follow-up rates varied between centres depending on participation in extended follow-up studies (REQUITEplus/RADprecise) (Fig. 1). Survival was not systematically collected over the follow-up period.

At baseline before the start of radiotherapy, the median age of participants was 58 years. Over 94% of participants self-identified as White (Supplementary Table 1).

### 3.2 Clinician-reported outcomes (CTCAE)

Of the 2056 patients, 1062 received normofractionation (≤2.2 Gy per fraction) and 994 received hypofractionation (>2.2 Gy per fraction). Boost irradiation was administered to 81% (859/1062) of patients in the normofractionation group (≤2.2 Gy per fraction) compared to 54% (535/994) in the hypofractionation group (Table 1). Table 2 and Fig. 2 present CTCAE v4 data stratified by fractionation schedule over time, shown as absolute patient numbers; corresponding relative (percentage) values are provided in Supplementary Fig. 1. Data completeness was high through two years but declined substantially thereafter (Fig. 1). Results are presented through five years post-radiotherapy where data are available; interpretation of late timepoints is limited by reduced sample sizes. Complete longitudinal data for all timepoints are shown in Table 2.

### 3.3 Acute side-effects

Erythema of any grade occurred at the end of radiotherapy in 89% of patients in the normofractionation group and 83% of patients in the hypofractionation group, and was the most frequently observed acute effect, with moderate to severe erythema (grade ≥ 2) representing the clinically relevant subset. Grade 2 erythema was highest at end of radiotherapy (normofractionation: 29% (305/1058), hypofractionation: 16% (157/992)). Grade 3 erythema was more common in the normofractionation group (2.3% (24/1058)) than in the hypofractionation group (0.4% (4/992)). Erythema decreased substantially at subsequent follow-up timepoints, with only isolated grade 2 cases observed.

The distribution of pain grades differed between fractionation schedules. In the normofractionation group, moderate to severe pain (grade ≥ 2) was seen in 11% of patients (115/1058) at end of radiotherapy, decreasing to 8% (80/997) at one year. The hypofractionation group showed 4.3% (42/992) at end of radiotherapy and 2.7% (24/876) at one year.

The incidence of skin ulceration was highest immediately after end of radiotherapy and was more common in the normofractionation group (grade 2: 3.7% (39/1058) vs. 0.7% (7/992); grade 3: 1.1% (12/1058) vs. 0.1% (1/992)).

Skin hyperpigmentation (grade 2) followed a similar temporal pattern, with highest rates at end of radiotherapy (normofractionation: 1.5% (16/1058), hypofractionation: 1.1% (11/992)). No grade 3 hyperpigmentation occurred.

Moderate to severe oedema (grade ≥ 2) was highest one year after end of radiotherapy in both groups (normofractionation: 3.8% (38/997), hypofractionation: 2.4% (21/876)), with lower proportions at subsequent timepoints. Grade 3 oedema was rare, with minimal cases in the normofractionation group and virtually none in the hypofractionation group.

### 3.4 Late side-effects

For late toxicity assessment, sample sizes decreased over time (Fig. 1). For each endpoint, the timepoint with the highest observed proportion within the five-year follow-up period is reported, with the focus being on clinically relevant moderate to severe effects (grade ≥ 2). Given variable follow-up completeness, percentages should be interpreted alongside absolute patient numbers.

Skin induration (fibrosis) within the tumour bed was the most frequently reported late side effect. The highest observed proportion was at one year after end of radiotherapy in the normofractionation group (8.6% (85/997)) and at four years in the hypofractionation group (7.8% (15/265 outside the tumour bed, the highest observed proportions were similarly at one year for normofractionation (2.7% (27/997)) and four years for hypofractionation (3.1% (6/265)).

Breast atrophy (grade ≥ 2) was already present at baseline in 5.4% (58/1062) of normofractionation patients and 3.4% (34/994) of hypofractionation patients prior to radiotherapy. Pre-existing effects are likely attributable to surgical intervention rather than radiotherapy, and therefore baseline toxicity burden complicates attribution of subsequent changes exclusively to radiotherapy, see Discussion. Rates were highest at two years in normofractionation (10.5% (104/953)) and three years in hypofractionation (16.8% (42/293)).

Moderate nipple retraction (grade 2) affected 1.4% of normofractionation patients (14/997) and 1.1% of hypofractionation patients (10/994) at one year after end of radiotherapy, with no grade 3 cases throughout follow-up.

Telangiectasia (grade 1) was highest at four years in both groups (7.9%); grade 2 was rare, with a maximum of 1% at four years, similarly within and outside the tumour bed. No grade 3 cases were observed.

Arm lymphoedema (grade 1) was highest at two years in the normofractionation group (4.5%, 43/953) and at four years in the hypofractionation group (3.7%, 7/190); grade 2 was highest at four years in the normofractionation group (1.8% (3/168)) and at three years in the hypofractionation group (1.2% (3/293)). Severe lymphoedema (grade 3) was observed in one patient (normofractionation group, three years post-radiotherapy).

Radiation-induced pneumonitis was infrequent, occurring in less than 1% of patients at any assessed timepoint.

### 3.5 Patient-reported outcomes

Patient-reported outcomes were prospectively assessed using the EORTC QLQ-C30 (Table 3) and breast cancer-specific QLQ-BR23 (Table 4) questionnaires for the overall cohort, without stratification by fractionation schedule. Response rates were 98.4% (*n* = 2025) at baseline and 84.8% (*n* = 1745) at 1 year, declining progressively to 1.7% (*n* = 34) by year 8.

The median overall quality of life (QLQ-C30) score was 67 (Q1–Q3: 50–83) at baseline and 83 (Q1–Q3: 67–92) among respondents assessed at 5–8 years. Median role functioning and social functioning scores were lower at the end of radiotherapy than at baseline and were higher among respondents assessed at 5–8 years than at baseline. Median physical, emotional, and cognitive functioning scores were similar across timepoints.

The median fatigue score was 33 (Q1–Q3: 11–44) at baseline and 22 (Q1–Q3: 11–44) at one year after the end of radiotherapy, with similar median scores at later timepoints. Other symptom scales including nausea/vomiting, pain, dyspnoea, insomnia, appetite loss, constipation, and diarrhea showed minimal changes in scores throughout the observation period.

The median breast-specific symptoms (QLQ-BR23) score was 17 (Q1–Q3: 8–25) at baseline and 33 (Q1–Q3: 17–50) at the end of radiotherapy, with scores at one year like baseline values. Arm symptoms scores were 11 (Q1–Q3: 0–22) at baseline and at two years, and 0 thereafter (Q1–Q3: 0–22 at 3–5 years; 0–11 at 6–8 years). Body image scores were 92 (Q1–Q3: 67–100) at baseline, 83 (Q1–Q3: 67–100) at the end of radiotherapy, and 100 (Q1–Q3: 75–100) from year 1 to 8. Sexual functioning scores were 17 (Q1–Q3: 0–33) at baseline and at the end of radiotherapy, and 33 (Q1–Q3: 0–33) from one year after the end of radiotherapy up to 8 years. Sexual enjoyment scores were 67 (Q1–Q3: 33–100) at all timepoints; these data had higher rates of missing values and should be interpreted with caution. Future perspective scores were 67 (Q1–Q3: 33–100) and the median systemic therapy side effects score was 14 (Q1–Q3: 5–24) across the observation period.

## 4 Discussion

### 4.1 Study context and data characteristics

The REQUITE study, along with the subsequent REQUITEplus and RADprecise studies, systematically collected standardised data on radiotherapy-related adverse effects in 2057 breast cancer patients across 27 centres in seven countries. This multi-national cohort represents one of the largest prospective datasets combining detailed CTCAE assessment, quality of life measures, genetic biomarkers, and radiotherapy physics data with follow-up extending to eight years. Data completeness was high in the first two years after end of radiotherapy but then decreased due to loss of follow-up in REQUITEplus and RADprecise.

### 4.2 Key findings in context

Our data for normofractionation (≤2.2 Gy per fraction) and hypofractionation (>2.2 Gy per fraction) in breast cancer patients provide comprehensive data on side-effects broadly consistent with data published in randomised trials. Acute adverse effects peaked at end of radiotherapy, with erythema documented in 83–89% of patients. Grade 2–3 acute effects, including pain and skin ulceration, were more frequently observed in the normofractionation group than in the hypofractionation group. Late normal-tissue effects were predominantly grade 1–2, with skin induration the most frequent event and breast atrophy reaching the highest observed grade ≥ 2 proportion (16.8%); grade 3 events were rare across all endpoints. These late effects were also assessed in the original UK START A and START B trials using patient self-assessment and photographic comparison; moderate or marked breast hardness by 5 years was reported in 42.6% of patients after 50 Gy in 25 fractions compared with 44.6% after 41.6 Gy and 34.9% after 39 Gy in 13 fractions in START A, and 42.3% after 50 Gy compared with 38.2% after 40 Gy in 15 fractions in START B [12], [13]. The Canadian hypofractionated trial reported favourable long-term local control and cosmetic outcome with 42.5 Gy in 16 fractions [14]. The IMPORT LOW trial [15], reported late normal-tissue effects only; patient-reported breast hardness at 5 years was lower after partial-breast and reduced-dose radiotherapy than after whole-breast radiotherapy, though this trial evaluated partial-breast irradiation, which was excluded from the present cohort, limiting direct comparability. IMPORT HIGH did not assess acute toxicity; at 5 years, moderate or marked breast induration was similar between standard-dose (48 Gy) simultaneous integrated boost and sequential boost but was higher with dose-escalated (53 Gy) boost [16]. The FAST-Forward acute toxicity sub-study, investigating ultra-hypofractionation (26–27 Gy in 5 fractions), reported lower rates of grade 3 acute skin toxicity compared with standard fractionation (40 Gy in 15 fractions) [17]. These trials used a range of assessment methods, including RTOG grading, patient-reported and photographic assessment, which are not necessarily equivalent to the CTCAE-based endpoints used in the present study; direct comparisons should therefore be interpreted with caution.

Patient-reported outcome scores were highest at the end of radiotherapy relative to other timepoints. Detailed individual patient trajectories are described in [18], [19]. Median breast-specific symptom scores were highest at the end of radiotherapy and like baseline values within two years. Median global health status, role functioning and social functioning scores were higher among respondents assessed at 5–8 years than at baseline (differences of 16, 17 and 17 points respectively). However, response rates declined to 1.7% (*n* = 34) by year 8, and these cross-sectional differences may reflect selective attrition and healthy survivor bias rather than within-patient change.

Our observational findings across heterogeneous treatment schedules, countries, and sites are broadly consistent with the START, Canadian, FAST-Forward, and IMPORT trials. Findings beyond five years are exploratory, given centre-dependent follow-up and a changing assessed population, and should be regarded as hypothesis-generating rather than conclusive.

### 4.3 Limitations

Substantial data attrition occurred over time. CTCAE data were available for only 48% (979/2057) of patients at five years or beyond, and EORTC QLQ-C30/BR23 response rates declined to 39.5% (*n* = 813) over the same period. The COVID-19 pandemic (2019–2022) considerably affected patient follow-up. Patients recruited at Mount Sinai Hospital in New York did not participate in extended follow-up beyond two years, representing planned centre non-participation; the centre in Ghent, by contrast, continued to participate but discontinued data collection after five years, representing loss to follow-up.

Differential attrition between treatment groups may reflect centre-specific participation patterns in extended follow-up studies.

Pre-existing adverse effects, particularly atrophy, were observed at baseline prior to radiotherapy, likely attributable to surgical intervention. This baseline toxicity burden complicates the attribution of subsequent changes exclusively to radiation therapy and may limit assessment of radiation-specific effects independent of surgical effects. Similarly, arm lymphoedema rates were not stratified by extent of axillary surgery (sentinel lymph node biopsy versus axillary lymph node dissection) or regional nodal irradiation in the current analysis. Axillary lymph node dissection is a well-established independent risk factor for lymphoedema [18], and distinguishing surgical from radiation-induced effects requires stratified analysis. This represents an important question for future investigation.

The lack of systematic vital status collection prevents comprehensive survival analysis, though breast cancer's generally favourable prognosis means this limitation is less significant [19]. Additionally, while our multi-national design enhances generalisability, heterogeneity in radiation techniques and dose prescriptions within the broad ≤2.2 Gy versus >2.2 Gy categorisation may obscure dose-specific effects. Furthermore, fractionation schedules were not evenly distributed across centres, with normofractionation predominantly used in Germany and Southern Europe and hypofractionation in Belgium and the UK, potentially confounding treatment effects with centre-specific practices.

### 4.4 Unique dataset characteristics and research opportunities

The REQUITE dataset offers substantial opportunities for secondary research into radiotherapy outcomes. For each patient in the study, detailed adverse effects and quality of life data were collected prospectively over long follow-up periods with comprehensively documented treatment information across a range of radiotherapy regimens, including different international patterns of practice. This creates possibilities for analyses that single-centre studies or protocol-restricted trials simply cannot do.

The data will allow researchers to address many questions we have not covered here. However, this paper establishes baseline adverse effects rates and temporal patterns as a starting point. More sophisticated analytical approaches could build on this foundation (www.requite.eu). Making these data available to the research community could help multiple groups work towards optimising radiotherapy and improving patient outcomes.

## 5 Conclusion

This comprehensive descriptive analysis of 2057 breast cancer patients provides detailed characterisation of acute and late side-effect patterns following normofractionation and hypofractionation. Our descriptive findings are broadly consistent with those of published randomised trials in the context of the evolving role of hypofractionation as the standard of care in many guidelines. The multi-national REQUITE dataset, combining detailed side-effect assessment, quality of life measures, radiotherapy physics data, and biological samples with extended follow-up, represents a valuable resource for secondary research into radiotherapy outcomes. We have established baseline adverse effects rates and temporal patterns as a reference framework. Researchers are encouraged to leverage this dataset's unique characteristics to address questions about dosimetric predictors, genetic associations [20], treatment interactions, and personalised approaches to optimising the therapeutic ratio in breast radiotherapy.

## Additional REQUITE and/or RADprecise/REQUITEplus Consortium Collaborators

Piet Ost, Yolande Lievens, Renée Bultijnck, Pieter Deseyne, Fréderic Duprez, Marc van Eijkeren, Elhaseen Elhamin, Valérie Fonteyne, Annick Van Greveling, Marcus Mareel, Christel Monten, Wilfried De Neve, Leen Paelinck, Giselle Post, Martijn Swimberghe, Katrien Vandecasteele, Tom Vercauteren

*Department of Radiation Oncology, Ghent University Hospital, Belgium*

Rita Aerts, Soumia Arredouani, Gilles Defraene, Erik van Limberghen, Stephanie Peeters, Caroline Weltens

*Department of Radiation Oncology, University Hospitals Leuven, Belgium*

Ben Vanneste

*Maastricht University Medical Center, Department of Radiation Oncology (Maastro clinic), Maastricht, The Netherlands*

Celine Bourgier, Muriel Brengues, Roxana Draghici

*Department of Radiation Oncology, University Federation of Radiation Oncology, Montpellier Cancer Institute, Univ Montpellier MUSE, Grant INCa_Inserm_DGOS_12553, Inserm U1194, Montpellier, France*

Francoise Bons

*Department of Radiation Oncology, University Federation of Radiation Oncology, Institut de Cancérologie du Gard, Nimes, France*

Thomas Blaschke

*Praxis für Strahlentherapie, Baden-Baden, Germany*

Christian Weiß

*Klinikum Darmstadt, Institut für Radionkologie und Strahlentherapie, Darmstadt, Germany*

Irmgard Helmbold, Anusha Müller

*Division of Cancer Epidemiology, German Cancer Research Center (DKFZ), Heidelberg, Germany*

Christian Weißenberger, Petra Stegmaier

*Zentrum für Strahlentherapie, Karlsruhe, Germany*

Johannes Claßen, Ulrich Giesche

*Klinik für Strahlentherapie, St. Vincentius-Kliniken gAG, Karlsruhe, Germany*

Marie-Luise Sautter-Bihl, Burkhard Neu

*Klinik für Radioonkologie und Strahlentherapie, Städtisches Klinikum, Karlsruhe, Germany*

Thomas Schnabel

*Klinik für Strahlentherapie und Radiologische Onkologie, Klinikum der Stadt Ludwigshafen gGmbH, Ludwigshafen, Germany*

Michael Ehmann, Benjamin Gauter-Fleckenstein, Frank Giordano

*Department of Radiation Oncology, Universitätsmedizin Mannheim, Medical Faculty Mannheim, Heidelberg University, Mannheim, Germany*

Jörg Schäfer

*Strahlentherapie Speyer, Germany*

Alessandro Cicchetti, Francesco Pisani

*Data Science Unit, Fondazione IRCCS Istituto Nazionale dei Tumori, Milan, Italy*

Tommaso Giandini

*Medical Physics Unit, Fondazione IRCCS Istituto Nazionale dei Tumori, Milan, Italy*

Marzia Franceschini

*Department of Radiation Oncology 2, Fondazione IRCCS Istituto Nazionale dei Tumori, Milan, Italy*

Claudia Sangalli, Sara Morlino, Laura Lozza, Maria C. De Santis, Riccardo Valdagni

*Department of Radiation Oncology 1, Fondazione IRCCS Istituto Nazionale dei Tumori, Milan, Italy*

Gabriele Pietro, Elena Delmastro, Elisabetta Garibaldi

*Department of Radiation Oncology, Fondazione del Piemonte per l'Oncologia Candiolo Cancer Institute, Candiolo (TO), Italy*

Manuel Altabas, Alba Delgado, Noelia Docampo, Alexandra Giraldo, Susana Magriña, Meritxell Mollà, Bibiana Piqué-Leiva, Mónica Ramos, Victoria M Reyes.

*Radiation Oncology Department, Vall d'Hebron Hospital Universitari, Vall d'Hebron Barcelona Hospital Campus, Barcelona, Spain*

Alejandro Seoane

*Medical Physics Department, Vall d'Hebron Hospital Universitari, Vall d'Hebron Barcelona Hospital Campus, Barcelona, Spain*

Olivia Fuentes-Rios, Ana Crujeiras González, Belinda Rodríguez-Lage, Juan Fernández-Tajes

*Fundación Pública Galega de Medicina Xenómica, Santiago de Compostela, Spain.*

*Instituto de Investigación Sanitaria de Santiago de Compostela, Spain.*

Patricia Calvo-Crespo, Monserrat Miranda Currás, Begoña Taboada-Valladares, Irene Fajardo-Paneque, Paloma Sosa-Fajardo, Laura Torrado-Moya, Isabel Dominguez-Rios

*Department of Radiation Oncology, Hospital Clínico Universitario de Santiago de Compostela, Servizo Galego de Saúde (SERGAS), Santiago de Compostela, Spain*

Ramón Lobato-Busto

*Radiophysics and Radiobiological Protection Service, Hospital Clínico Universitario de Santiago de Compostela, Servizo Galego de Saúde (SERGAS), Santiago de Compostela, Spain*

Victoria Harrop

*Queen Elizabeth Hospital, University Hospitals Birmingham NHS Trust, Birmingham, United Kingdom*

Debbie Payne

*Centre for Integrated Genomic Medical Research (CIGMR), Manchester, United Kingdom*

Manjusha Keni

*Department of Oncology, Derby Teaching Hospitals NHS Foundation Trust, Derby, United Kingdom*

Hannah Dobbelaere, Kerstie Johnson, Samuel Lavers

*Department of Genetics and Genome Biology, Leicester Cancer Research Centre, University of Leicester, Leicester, UK*

Simon Wright, Sridhar Thiagarajan, Luis Aznar-Garcia, Kiran Kancherla, Christopher Kent, Subramaniam Vasanthan, Ion Bioangiu, Kufre Sampson, Ahmed Osman

*Department of Oncology, Leicester Royal Infirmary, University Hospitals of Leicester NHS Trust, Leicester, United Kingdom*

Donna Appleton, Monika Kaushik, Frances Kenny, Hazem Khout, Jaroslaw Krupa, Kelly V. Lambert, Simon Pilgrim, Sheila Shokuhi, Kalliope Valassiadou,

*Department of Breast Surgery, Glenfield Hospital, University Hospitals of Leicester NHS Trust, Leicester, United Kingdom*

Corinne Faivre-Finn

*Division of Cancer Sciences, University of Manchester, Manchester, United Kingdom*

Karen Foweraker, Abigail Pascoe, Claire P. Esler

*City Hospital, Nottingham University Hospitals NHS Trust, Nottingham, United Kingdom*

Daniel S. Higginson

*Department of Radiation Oncology, Memorial Sloan Kettering Cancer Center, New York, NY, United States*

Sheryl Green, Ava Golchin, Richard Stock

*Icahn School of Medicine at Mount Sinai, New York, NY, United States*

## Declaration of generative AI and AI-assisted technologies in the manuscript preparation process

During the preparation of this work, the authors used Claude (Anthropic) to improve the language and readability of the English text. After using this tool, the authors reviewed and edited the content as needed and take full responsibility for the content of the published article.

## Author contributions

**Conceptualization**: Tina Bockstiegel, Adam Webb, Tim Rattay, Elena Sperk; **Data collection/Data management**: Tina Bockstiegel, Adam Webb, Miguel E Aguado-Barrera, Ester Aguado-Flor, Manuel Altabas, Marianne Aznar, David Azria, Renée Bultijnck, Jenny Chang-Claude, Alessandro Cicchetti, Maria Carmen De Santis, Alison Dunning, Laura Fachal, Olivia Fuentes-Rios, Alexandra Giraldo, Antonio Gómez-Caamaño, Sara Gutiérrez-Enríquez, Carsten Herskind, Philipp Heumann, Kerstie Johnson, Rudolf Kaaks, Sarah L. Kerns, Maarten Lambrecht, Eliana La Rocca, Meritxell Mollà, Tiziana Rancati, Victoria M Reyes, Juan Camilo Rosas Romero, Barry S. Rosenstein, Dirk De Ruysscher, Petra Seibold, Rebecca M. Shearer, R. Paul Symonds, Begoña Taboada-Valladares, Christopher J. Talbot, Ana Vega, Liv Veldeman, Marlon R. Veldwijk, Catharine M. L. West, Tim Rattay, Elena Sperk; **Formal analysis**: Tina Bockstiegel, Adam Webb, Elena Sperk, Tim Rattay; **Funding acquisition**: Jenny Chang-Claude, Christopher J. Talbot, Catharine M. L. West; **Methodology**: Tina Bockstiegel, Adam Webb, Tim Rattay, Elena Sperk; **Patient advocates**: Erik Briers, Tim Ward, Hilary Stobart; **Project administration**: Petra Seibold; **Supervision**: Elena Sperk, Tim Rattay; **Visualization**: Tina Bockstiegel, Adam Webb; **Roles/Writing – original draft**: Tina Bockstiegel, Tim Rattay, Elena Sperk, Adam Webb; **Writing – review & editing**: Tina Bockstiegel, Adam Webb, Miguel E Aguado-Barrera, Ester Aguado-Flor, Manuel Altabas, Marianne Aznar, David Azria, Renée Bultijnck, Jenny Chang-Claude, Alessandro Cicchetti, Maria Carmen De Santis, Alison Dunning, Laura Fachal, Olivia Fuentes-Rios, Alexandra Giraldo, Antonio Gómez-Caamaño, Sara Gutiérrez-Enríquez, Carsten Herskind, Philipp Heumann, Kerstie Johnson, Rudolf Kaaks, Sarah L. Kerns, Maarten Lambrecht, Eliana La Rocca, Meritxell Mollà, Tiziana Rancati, Victoria M Reyes, Juan Camilo Rosas Romero, Barry S. Rosenstein, Dirk De Ruysscher, Petra Seibold, Rebecca M. Shearer, R. Paul Symonds, Begoña Taboada-Valladares, Christopher J. Talbot, Ana Vega, Liv Veldeman, Marlon R. Veldwijk, Catharine M. L. West, Tim Rattay, Elena Sperk. All authors have approved the final manuscript

## Declaration of competing interest

The authors declare the following financial interests/personal relationships which may be considered as potential competing interests: D. Azria was involved in the creation of the start-up NovaGray in 2015. D. De Ruysscher: none related to the current manuscript. Outside the current manuscript: advisory board of Astra Zeneca, Bristol-Myers-Squibb, Roche/Genentech, Merck/Pfizer, Celgene, Noxxon, Mologen and has received investigator initiated grants from Bristol-Myers-Squibb, Boehringer Ingelheim and Astra-Zeneca. E. Sperk: none related to the current manuscript. Outside the current manuscript: General speakers bureau Zeiss Meditec, travel support Zeiss Meditec. The other authors declare that they do not have any conflict of interest.

## Appendix A Supplementary data

Supplementary material 1 Supplementary Table 1: Patient characteristics at baseline before the start of radiotherapy, 2 years later and beyond 5 years overall

Supplementary material 2 Supplementary Figure 1: Endpoints - CTCAE symptoms by follow-up timepoints, stratified by fraction size (≤ 2.2 Gy vs. > 2.2 Gy) in relative frequencies
