# Supplementary material for: Early and late CTCAE and patient-reported outcomes following breast radiotherapy in the REQUITE cohort
Source: Clin Transl Radiat Oncol. 2026 Oct 7;62:101307. doi: 10.1016/j.ctro.2026.101307 (PMC13696215; doi:10.1016/j.ctro.2026.101307)
Supplement: Supplementary file 1 [file mmc1.docx]

Supplementary Table 1: Baseline characteristics of REQUITE and RADprecise breast cancer patients prior to radiotherapy and remaining in the cohort at 2 years and 5 years after the end of radiotherapy

|  | Pre-RT | 2 years | 5+ years^6^ |
| --- | --- | --- | --- |
| **Characteristic** | **N = 2,050**^1^ | **N = 1,759**^1^ | **N = 979**^1^ |
| Country |  |  |  |
| Belgium | 550 (27%) | 487 (28%) | 191 (20%) |
| France | 449 (22%) | 409 (23%) | 384 (39%) |
| Germany | 212 (10%) | 184 (10%) | 55 (5.6%) |
| Italy | 101 (4.9%) | 89 (5.1%) | 78 (8.0%) |
| Spain | 315 (15%) | 284 (16%) | 141 (14%) |
| United Kingdom | 347 (17%) | 238 (14%) | 130 (13%) |
| United States | 76 (3.7%) | 68 (3.9%) |  |
| Age (years) | 58 (50, 66) | 58 (50, 66) | 57 (50, 65) |
| Weight at cancer diagnosis (kg) | 68 (60, 78) | 67 (60, 78) | 67 (59, 76) |
| Unknown | 14 | 10 | 4 |
| Height (cm) | 163 (158, 168) | 163 (158, 168) | 162 (158, 167) |
| Unknown | 10 | 9 | 1 |
| Smoker |  |  |  |
| Never | 1,151 (57%) | 1,010 (58%) | 550 (56%) |
| Ex before cancer diagnosis | 513 (25%) | 435 (25%) | 255 (26%) |
| Ex since cancer diagnosis | 83 (4.1%) | 67 (3.9%) | 38 (3.9%) |
| Current | 281 (14%) | 228 (13%) | 133 (14%) |
| Unknown | 22 | 19 | 3 |
| Ethnicity |  |  |  |
| White (European or American European) | 1,927 (94%) | 1,657 (94%) | 939 (96%) |
| Other | 117 (5.7%) | 98 (5.6%) | 39 (4.0%) |
| Unknown | 6 | 4 | 1 |
| BMI^2^ (kg/m^2^) | 25.5 (22.6, 29.3) | 25.4 (22.6, 29.1) | 25.3 (22.5, 28.7) |
| Unknown | 20 | 16 | 5 |
| Num nodes involved |  |  |  |
| 0 | 1,469 (79%) | 1,258 (79%) | 721 (81%) |
| 1 | 227 (12%) | 194 (12%) | 96 (11%) |
| 2 | 75 (4.0%) | 66 (4.1%) | 37 (4.1%) |
| 3+ | 93 (5.0%) | 83 (5.2%) | 38 (4.3%) |
| Unknown | 186 | 158 | 87 |
| Oestrogen receptor status |  |  |  |
| Positive (>10%) | 1,662 (86%) | 1,432 (87%) | 779 (88%) |
| Negative (<=10%) | 264 (14%) | 222 (13%) | 108 (12%) |
| Unknown | 124 | 105 | 92 |
| HER2 status |  |  |  |
| Positive | 205 (12%) | 181 (12%) | 95 (11%) |
| Negative | 1,575 (88%) | 1,343 (88%) | 743 (89%) |
| Unknown | 270 | 235 | 141 |
| Radiotherapy IMRT^3^ |  |  |  |
| No | 1,032 (50%) | 928 (53%) | 601 (62%) |
| Yes | 1,015 (50%) | 829 (47%) | 376 (38%) |
| Unknown | 3 | 2 | 2 |
| Radiotherapy treatment position |  |  |  |
| Prone | 299 (15%) | 244 (14%) | 141 (14%) |
| Supine | 1,750 (85%) | 1,514 (86%) | 837 (86%) |
| Unknown | 1 | 1 | 1 |
| Radiotherapy boost |  |  |  |
| No | 657 (32%) | 507 (29%) | 280 (29%) |
| Yes | 1,393 (68%) | 1,252 (71%) | 699 (71%) |
| Radiotherapy boost sequence |  |  |  |
| Sequential including IORT^4^ boost | 1,137 (82%) | 1,027 (82%) | 589 (84%) |
| Simultaneous integrated | 216 (16%) | 194 (15%) | 104 (15%) |
| Simultaneous integrated and sequential^5^ | 40 (2.9%) | 31 (2.5%) | 6 (0.9%) |
| Unknown | 657 | 507 | 280 |
| Radiotherapy breast dose (Gy) | 50.0 (40.1, 50.0) | 50.0 (40.1, 50.0) | 50.0 (40.1, 50.0) |
| Dose per fraction |  |  |  |
| <= 2.2 Gy | 1,058 (52%) | 953 (54%) | 590 (60%) |
| 2.2 - 3 Gy | 936 (46%) | 758 (43%) | 369 (38%) |
| > 3 Gy | 56 (2.7%) | 48 (2.7%) | 20 (2.0%) |
| Tumour side of primary |  |  |  |
| Left | 1,044 (51%) | 902 (51%) | 507 (52%) |
| Right | 994 (49%) | 850 (49%) | 463 (48%) |
| Unknown | 12 | 7 | 9 |
| Pathological tumour size (mm) | 14 (9, 20) | 14 (9, 20) | 14 (9, 19) |
| Unknown | 73 | 59 | 37 |
| Pathologic UICC T-stage |  |  |  |
| Tis | 252 (13%) | 218 (13%) | 127 (14%) |
| T1 | 1,357 (68%) | 1,157 (68%) | 649 (69%) |
| T2 | 332 (17%) | 284 (17%) | 140 (15%) |
| T3 | 8 (0.4%) | 7 (0.4%) | 3 (0.3%) |
| T4 | 5 (0.3%) | 5 (0.3%) | 2 (0.2%) |
| TX | 36 (1.8%) | 33 (1.9%) | 18 (1.9%) |
| Unknown | 60 | 55 | 40 |
| Neo-adjuvant UICC T-stage |  |  |  |
| Tis | 3 (1.4%) | 3 (1.6%) | 0 (0%) |
| T1 | 63 (30%) | 58 (31%) | 25 (32%) |
| T2 | 128 (62%) | 115 (61%) | 48 (62%) |
| T3 | 9 (4.3%) | 7 (3.7%) | 4 (5.1%) |
| TX | 5 (2.4%) | 4 (2.1%) | 1 (1.3%) |
| Unknown | 1,842 | 1,572 | 901 |
| Adjuvant chemotherapy |  |  |  |
| No | 1,579 (77%) | 1,349 (77%) | 738 (76%) |
| Yes | 460 (23%) | 403 (23%) | 233 (24%) |
| Unknown | 11 | 7 | 8 |
| Neo-adjuvant chemotherapy |  |  |  |
| No | 1,849 (91%) | 1,581 (90%) | 895 (92%) |
| Yes | 190 (9.3%) | 171 (9.8%) | 76 (7.8%) |
| Unknown | 11 | 7 | 8 |
| Endocrine therapy |  |  |  |
| Tamoxifen | 760 | 639 | 342 |
| Aromatase inhibitor | 743 | 644 | 376 |
| Tamoxifen + Aromatase inhibitor | 63 | 59 | 14 |
| Neither | 57 | 55 | 26 |
| Unknown | 431 | 360 | 221 |
| Radiotherapy to supraclavicular fossa |  |  |  |
| No | 1,773 (87%) | 1,509 (86%) | 849 (87%) |
| Yes | 262 (13%) | 237 (14%) | 125 (13%) |
| Unknown | 15 | 13 | 5 |
| Radiotherapy axilla levels |  |  |  |
| None | 1,785 (88%) | 1,532 (88%) | 872 (90%) |
| I | 13 (0.6%) | 12 (0.7%) | 8 (0.8%) |
| II | 15 (0.7%) | 12 (0.7%) | 5 (0.5%) |
| III | 88 (4.3%) | 81 (4.6%) | 36 (3.7%) |
| I-III | 62 (3.1%) | 48 (2.8%) | 25 (2.6%) |
| Other | 66 (3.3%) | 57 (3.3%) | 26 (2.7%) |
| Unknown | 21 | 17 | 7 |
| Breast surgery type |  |  |  |
| Segmentectomy/Quadrantectomy | 1,112 (55%) | 999 (57%) | 625 (65%) |
| Wide local excision | 917 (45%) | 745 (43%) | 341 (35%) |
| Unknown | 21 | 15 | 13 |
| Axillary surgery type |  |  |  |
| Sentinel node biopsy | 1,524 (81%) | 1,295 (80%) | 731 (81%) |
| Planned axillary dissection | 158 (8.4%) | 136 (8.4%) | 66 (7.3%) |
| Sentinel node biopsy plus axillary dissection | 202 (11%) | 188 (12%) | 104 (12%) |
| Unknown | 166 | 140 | 78 |
| ^1^ n (%); Median (Q1, Q3)  ^2^ BMI: Body mass index  ^3^ IMRT: Intensity modulated radiation therapy  ^4^ IORT: Intraoperative radiation therapy  ^5^ WBI = whole-breast irradiation, 28 × 1.8 Gy to 50.4 Gy; SIB = simultaneous integrated boost, 28 × 0.2 Gy to 56 Gy; SEB = sequential external boost, 2 × 2 Gy to a maximum total dose of 60 Gy  ^6^ For the combined 5–8-year category, the maximum grade recorded for each patient within this period is shown  Percentages are calculated using the number of patients with available data as the denominator and therefore sum to 100% across the reported categories. Patients with unknown or missing data are reported as absolute numbers only. | | | |
