# Supplementary material for: Early and late CTCAE and patient-reported outcomes following breast radiotherapy in the REQUITE cohort
Source: Clin Transl Radiat Oncol. 2026 Oct 7;62:101307. doi: 10.1016/j.ctro.2026.101307 (PMC13696215; doi:10.1016/j.ctro.2026.101307)
Supplement: Supplementary file 2 [file mmc2.docx]

**Supplementary Figure 1:** Endpoints - CTCAE symptoms by follow-up timepoints, stratified by fraction size (≤ 2.2 Gy vs. > 2.2 Gy) in relative frequencies

*Percentage of patients for each visit*

*For the combined 5–8-year category, the maximum grade recorded for each patient within this period is shown.*
